# Associations Between Healthy Lifestyle Trajectories and the Incidence of Cardiovascular Disease With All-Cause Mortality: A Large, Prospective, Chinese Cohort Study

**DOI:** 10.3389/fcvm.2021.790497

**Published:** 2021-12-20

**Authors:** Xiong Ding, Wei Fang, Xiaojie Yuan, Samuel Seery, Ying Wu, Shuohua Chen, Hui Zhou, Guodong Wang, Yun Li, Xiaodong Yuan, Shouling Wu

**Affiliations:** ^1^School of Public Health, North China University of Science and Technology, Tangshan, China; ^2^Shantou University Medical College, Shantou, China; ^3^Department of Epidemiology, School of Public Health, Air Force Medical University, Xi'an, China; ^4^Division of Health Research, Faculty of Health and Medicine, Lancaster University, Lancaster, United Kingdom; ^5^Department of Cardiology, Kailuan General Hospital, Tangshan, China; ^6^College of Nursing and Rehabilitation, North China University of Science and Technology, Tangshan, China; ^7^Department of Neurosurgery, Kailuan General Hospital, Tangshan, China

**Keywords:** lifestyle, cardiovascular disease, all-cause mortality, trajectory, BMI change, cohort

## Abstract

**Background:** Lifestyles generally change across the life course yet no prospective study has examined direct associations between healthy lifestyle trajectories and subsequent cardiovascular disease (CVD) or all-cause mortality risk.

**Methods:** Healthy lifestyle score trajectories during 2006–2007, 2008–2009, and 2010–2011 were collated through latent mixture modeling. An age-scale based Cox proportional hazard regression model was implemented to calculate hazard ratios (HR) with corresponding 95% confidence intervals (CI) for developing CVD or all-cause mortality across healthy lifestyle trajectories.

**Results:** 52,248 participants were included with four distinct trajectories identified according to healthy lifestyle scores over 6 years i.e., low-stable (*n* = 11,248), high-decreasing (*n* = 7,374), low-increasing (*n* = 7,828), and high-stable (*n* = 25,799). Compared with the low-stable trajectory, the high-stable trajectory negatively correlated with lower subsequent risk of developing CVD (HR, 0.73; 95% CI, 0.65–0.81), especially stroke (HR, 0.70; 95% CI, 0.62–0.79), and all-cause mortality (HR, 0.89; 95% CI, 0.80–0.99) under a multivariable-adjusted model. A protective effect for CVD events was observed only in men and in those without diabetes, while a reduced risk of all-cause mortality was observed only in those older than 60 years, though interactions were not statistically significant. Marginally significant interactions were observed between the changing body mass index (BMI) group, healthy lifestyle score trajectories and stratified analysis. This highlighted an inverse correlation between the high-stable trajectory and CVD in BMI decreased and stable participants as well as all-cause mortality in the stable BMI group. The low-increasing trajectory also had reduced risk of CVD only when BMI decreased and in all-cause mortality only when BMI was stable.

**Conclusions:** Maintaining a healthy lifestyle over 6 years corresponds with a 27% lower risk of CVD and an 11% lower risk in all-cause mortality, compared with those engaging in a consistently unhealthy lifestyle. The benefit of improving lifestyle could be gained only after BMI change is considered further. This study provides further evidence from China around maintaining/improving healthy lifestyles to prevent CVD and early death.

## Introduction

Cardiovascular disease (CVD) has a high global prevalence and remains the leading contributor to global mortality (1). Liu et al. conducted an exhaustive analysis of the burden of CVD in China and found that the prevalence of CVD doubled since 1990, reaching 94 million in 2016 (2). Aside from calculating that CVD is responsible for 42% of all-cause mortality (3), evidence also appeared to suggest that the burden of this disease maybe lower in coastal provinces where there is a higher level of economic development. However, diversity in China is not based solely upon regional differences nor should it be based upon geo-specific investment because lifestyles are culturally embedded, and there are employment-based migratory patterns which determine accessibility and health-seeking behaviors. Therefore, it remains necessary to develop the evidence-base around healthy lifestyles for public health interventions.

Aside from the established risk factors such as sex and age, chronic comorbidities, modifiable unhealthy lifestyle factors, including smoking, excessive alcohol intake, physical inactivity, sedentary behaviors and unhealthy diet, have proven to increase the risk of CVD and CVD-related mortality (4, 5). The converse is also true, with a higher number of healthy lifestyle factors correlating with significantly lower risk of developing CVD (6) and mortality (7, 8). Indeed, obesity (9), which is linked to type 2 diabetes (10) and hypertension (11), is considered a primary comorbidity of CVD. However, there is a distinct clinical iceberg when it comes to type 2 diabetes and hypertension where obese people teeter on the edge, in a pre-diabetic state, suffering only mild, and perhaps even infrequent symptoms. This means that aside from analyzing prevalence rates we must focus on lifestyles to develop public health interventions and to maximize the benefit.

Lifestyles however, often change across the life-course therefore retrospective, cross-sectional studies are often unable to disentangle the minutia involved in such change. That is, given the prospects of change, retrospectives have a tendency to assess non-causal explanations and can be constrained by data availability. Researchers in the field have managed to identify a number of key biological and lifestyle factors which correspond with an increased likelihood of developing CVD, as well as other types of diseases across three generations of participants. Yet, to the best of our knowledge, there has been no prospective study published which examines direct associations between healthy lifestyle trajectories and subsequent CVD or all-cause mortality risk in China.

## Materials and Methods

### Study Design and Participants

The Kailuan cohort was established in 2006 in Tangshan, Hebei province, China, where participants undergo a comprehensive check-up every two years. Please see The Kailuan Study, trial identification: ChiCTR-TNC-11001489; registration site: http://www.chictr.org.cn/index.aspx; registration number: 11001489 (12) for further details. Since June 2006, 101,510 adults have been enrolled including 81,110 men and 20,400 women. Participants were enrolled from 11 hospitals in the Kailuan community and completed questionnaires, clinical examinations, and laboratory tests. After excluding 7,953 participants i.e., 3,394 with CVD diagnosis and 4,559 who did not have sufficient data around lifestyles, 93,557 remained. We excluded a further 3,589 who developed CVD or died and 37,720 who did not complete questionnaires etc. after the second visit. Finally, 52,249 participants were included for analysis, please see the Supplementary Material, Figure 1 for further details. The Ethics Committees at Kailuan General Hospital approved this study and all participants were asked to provide informed consent before analysis took place.

**Figure 1:**
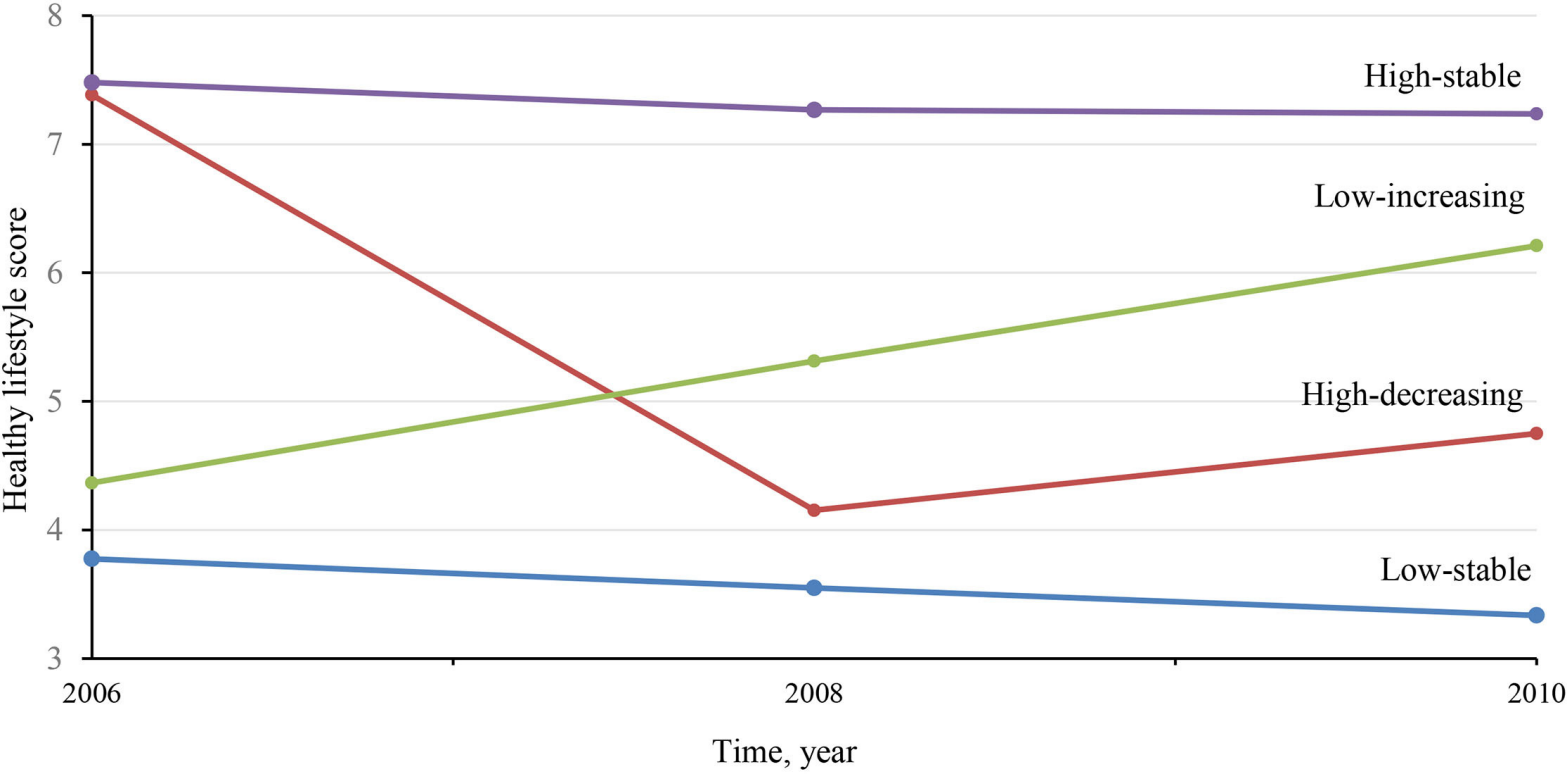
Mean healthy lifestyle score in 2006, 2008 and 2010, according to four healthy lifestyle score trajectories.

### Definitions of Lifestyles Status at Baseline

Information was gathered over three biennial visits. Data around lifestyle status, including: smoking status, alcohol intake, physical activity, sedentary behaviors and salt intake, were collected face-to-face by trained staff using a standard questionnaire, implemented in previous research (13). A score was assigned for each component of the five predefined lifestyles, with zero representing a poor level, intermediate represented by 1 and ideal was given 2 points. Please see Supplementary Table 1 for further details. Totals ranged from 0 which was considered the worst possible scenario and 10 considered optimal.

Current smokers were defined as participants who smoked at least one cigarette, every day for at least 6 months prior. While past smokers were defined as participants who reported smoking previously but quit smoking before or during the survey. Likewise, current drinkers were defined as participants who drank at least one time per month over the past year. Whereas, past drinkers were defined as those who reported having drank previously but had quit before or during the survey.

Poor, intermediate or an ideal amount of sedentary behavior was defined with an average sedentary time ≥8 h/day, 4–7 h/day and <4 h/day, respectively. Intermediate physical activity was assigned to those taking physical activity for 20+ min per time and 1–2 times per week. While an ideal level activity was defined as ≥3 times per week during leisure time.

Due to the lack of detailed dietary data between 2006–2010 and considering the influence of salt intake over CVD risks among the Chinese population, total salt intake was determined by questionnaire which can be considered a surrogate measure for diet quality. Poor, intermediate and ideal diet was defined with a salt intake ≥10 g/day, 6–9 g/day and <6 g/day. As described previously, a validation was made according 2014 dataset when diet information was collected through a semi-quantitative, validated food-frequency questionnaire, suggesting a strong association between higher perceived salt intake and lower healthy diet score (14).

### Covariates at Baseline

Demographics including age, sex, level of education, comorbidities and related medicaments, as well as laboratory tests were collected. Education was classified as less than high school and high school or above. Weight and height were measured, before calculating body mass index (BMI) as weight in kilograms divided by height in meters squared. Overweight/obesity was defined as BMI ≥ 24 kg/m^2^ which is in accordance with Chinese standards (15). Hypertension was defined as blood pressure ≥140/90 mmHg, administration of antihypertensive medication, or self-reported previous diagnosis. Diabetes mellitus was defined as a fasting blood glucose (FBG) at concentrations of ≥7.0 mmol/L, administration of glucose-lowering medication, or self-reported previous diagnosis. Dyslipidemia was defined as achieving one of the following criteria according to Chinese standards: (1) triglyceride (TG) ≥ 2.3 mmol/L; (2) total cholesterol (TC) ≥ 6.4 mmol/L; (3) low density lipoprotein-cholesterol (LDL-C) ≥ 4.1 mmol/L, or (4) high density lipoprotein cholesterol (HDL-C) <1.0 mmol/L (16).

### Ascertainment of Incident CVD Events and Death

The main outcome was incidence of CVD events including myocardial infarction and stroke. As previously described (17), all participants were linked to the Municipal Social Insurance Institution and the Hospital Discharge Register for incidence of CVD, which covers all of the Kailuan study participants. To further identify potential CVD events, we reviewed discharge lists from 11 hospitals during 2006–2019 and asked for a history of CVD via a questionnaire during biennial interviews.

For all suspected CVD events, three experienced clinical adjudicators, who were blinded to the study design, reviewed medical records. An incident myocardial infarction was diagnosed according to the World Health Organization's (WHO) Multinational Monitoring of Trends and Determinants in Cardiovascular Disease criteria on the basis of clinical symptoms and dynamic changes in cardiac enzymes and/or biomarker concentrations and electrocardiogram results (18). Stroke was diagnosed according to symptoms and neuroimages captured using computed tomographic (CT) or magnetic resonance imaging (MRI), with other diagnostic reports provided in accordance with WHO criteria (19), as described previously (20). All-cause mortality data was collected via provincial vital statistics offices.

### Statistical Analyses

Healthy lifestyle scores for each of the three visits across 2006–2007, 2008–2009, and 2010–2011 were computed separately. Trajectories were then determined using latent mixture modeling, within the PROC TRAJ procedure (21). Model fit was assessed using Bayesian information criterion and the number of participants in each trajectory i.e. >5% of the overall population.

The basic characteristics among trajectories were compared using one-way ANOVA or the Kruskal-Wallis H test for continuous variables and chi-square test for categorical variables. Person-time at follow-up was computed for each participant from the date of the third survey (which was the baseline) to the date of CVD diagnosis, death, or the end of follow-up on the 31^st^ December, 2019), whichever came first. The proportional hazards assumption was satisfied and an age-scale based Cox proportional hazard regression model was implemented to calculate the hazard ratios (HR) and 95% confidence intervals (CI) for developing CVD or all-cause mortality across healthy lifestyle score trajectories. A multivariable-adjusted model was then developed for sex, educational level, BMI, FBG, systolic blood pressure (SBP), estimated glomerular filtration rate (eGFR), LDL-C, and HDL-C. In order to ensure the association could not be explained by a single healthy lifestyle score during follow-up, we additionally adjusted for 2006 and 2010 healthy lifestyle score.

In addition, analyses were conducted using the incident of myocardial infarction and stroke, separately. In order to gain insight into a potential correlation between healthy lifestyle score trajectories and CVD or all-cause mortality, data were arranged and, in some instances stratified according to sex, age, overweight/obesity, hypertension, diabetes mellitus, dyslipidemia and BMI change. Potential interactions were assessed between healthy lifestyle score trajectories and factors after controlling for the aforementioned covariates. Stratified results of these factors are presented for consideration.

Sensitivity analyses were also conducted strengthen the connection. To ensure the association was not influenced by imputation, repeated analysis was conducted in the dataset without imputating. To strengthen the temporality of connections, lag-analysis was conducted by excluding CVD incidents or death, separately, with onset during the first 2 years of follow-up, which may not be caused by lifestyles in such a short time.

Missing data for covariates are presented in the Supplementary Table 2 and were imputed using Multivariate Imputation by Chained Equations (MICE) within the PROC MI procedure in SAS. All tests were two-sided with the threshold for statistical significance set at *p* < 0.05. All analyses were performed using SAS version 9.4 (SAS Institute, Inc, Cary, NC).

## Results

### Population Characteristics

52,248 participants including 39,793 men and 12,455 women were involved. The average age was 52.6 +/- 11.8 years. Please see Supplementary Figure 1 for further demographic summary data. Four distinct trajectories were identified according to healthy lifestyle scores over the 6 years (Figure 1). There was a low-stable trajectory which consisted of 11,248 participants with a mean healthy lifestyle score (mHLS) and ranged from 3.3–3.8 between 2006–2010. There was also a high-decreasing trajectory (*n* = 7,374, mean mHLS decreased from 7.4 in 2006 to 4.8 in 2010). We also observed a low-increasing trajectory (*n* = 7,828, mHLS increased from 4.4 in 2006 to 6.2 in 2010), and high-stable trajectory (*n* = 25,799, mHLS range 7.2–7.5 during 2006–2010).

Demographics within the four trajectories are provided in Table 1. Age, sex, level of education, medical history i.e., hypertension and diabetes, and biological data including BMI, SBP, diastolic blood pressure (DBP), FBG, eGFR, TG, LDL-C, and HDL-C were quite different among trajectories.

**Table 1:** Characteristics of 52,248 participants according to healthy lifestyle score trajectories from 2006 to 2010.

**Characteristics**	**Low-stable** **(*n* = 11,248)**	**High-decreasing** **(*n* = 7,374)**	**Low-increasing (*n* = 7,828)**	**High-stable** **(*n* = 25,798)**	***P* value**
Age (years)	49.1 ± 10.5	50.4 ± 10.5	51.9 ± 11.7	54.9 ± 12.2	<0.001
Men (%)	11,177 (99.4)	6,989 (94.8)	7,403 (94.6)	14,224 (55.1)	<0.001
High school education or above (%)	3,561 (31.7)	1,807 (24.5)	2,165 (27.7)	6,860 (26.6)	<0.001
BMI (kg/m^2^)	25.1 ± 3.3	25.2 ± 3.4	25.2 ± 3.3	25.0 ± 3.5	<0.001
SBP (mmHg)	129.7 ± 17.4	130.0 ± 17.6	131.9 ± 18.7	130.6 ± 20.3	<0.001
DBP (mmHg)	85.2 ± 10.6	85.3 ± 10.4	84.9 ± 10.7	83.3 ± 10.9	<0.001
FBG (mmol/L)	5.6 ± 1.4	5.6 ± 1.5	5.7 ± 1.5	5.6 ± 1.5	0.001
Hs-CRP (interquartile range, mg/L)	1.0 (1.8)	0.9 (2.1)	1.1 (2.0)	1.0 (1.9)	<0.001
eGFR (interquartile range, ml/min/1.73 m^2^)	101.1 (20.2)	90.8 (25.9)	95.7 (27.2)	87.9 (29.7)	<0.001
LDL-C (mmol/L)	2.6 ± 0.7	2.6 ± 0.8	2.7 ± 0.7	2.6 ± 0.8	<0.001
HDL-C (mmol/L)	1.6 ± 0.5	1.5 ± 0.4	1.5 ± 0.4	1.6 ± 0.4	<0.001
Hypertension (%)	6,378 (56.7)	4,439 (60.2)	4,441 (56.7)	14,353 (55.6)	<0.001
Diabetes mellitus (%)	1,286 (11.4)	937 (12.7)	1,026 (13.1)	3,509 (13.6)	<0.001
Using of antihypertensive agent (%)	4,371 (38.9)	3,389 (46.0)	3,285 (42.0)	11,266 (43.7)	<0.001
Use of hypoglycemic medication (%)	816 (7.3)	651 (8.8)	739 (9.4)	2,609 (10.1)	<0.001
Use of lipid-lowering medication (%)	203 (1.8)	88 (1.2)	168 (2.1)	443 (1.7)	<0.001

*BMI, body mass index; SBP, systolic blood pressure; DBP, diastolic blood pressure; FBG, fasting blood glucose; Hs-CRP, hypersensitive C-reactive protein; eGFR, estimated glomerular filtration rate; LDL-C, Low-density lipoprotein cholesterol; HDL, High-density lipoprotein cholesterol. Data are presented as n (%), mean ± SD or median (interquartile range) according to variable category. Pearson's chi-square test, one-way ANOVA analysis or Kruskal-Wallis test were used to compare differences between groups properly*.

### Healthy Lifestyle Score Trajectories and CVD Incidence and All-Cause Mortality

2,724 CVD events were observed over the 8 years follow-up period. As can be seen in Table 2, the incidence of low-stable, high-decreasing, low-increasing and high-stable trajectories was 6.23, 6.56, 7.10 and 5.58 per 1,000 person-years (PYs), respectively. Compared with low-stable trajectory, high-stable trajectory appeared to negatively correlate with a lower subsequent risk of developing CVD, with an HR of 0.63 (95% CI, 0.57–0.69) in unadjusted model and 0.73 (95% CI, 0.65–0.81) in the multivariable-adjusted model 1.

**Table 2:** Incidence of CVD and all-cause mortality according to healthy lifestyle score trajectories from 2006 to 2010.

	**Health lifestyle score trajectories, HR (95% CI)**			
	**Low-stable**	**High-decreasing**	**Low-increasing**	**High-stable**
CVD				
N/n	607/11,248	418/7,374	475/7,828	1,224/25,798
Incidence rate, per 1,000 person-years	6.23 (5.75–6.75)	6.56 (5.96–7.22)	7.10 (6.49–7.77)	5.58 (5.27–5.90)
Unadjusted model	Reference	0.97 (0.86–1.10)	0.94 (0.84–1.06)	0.63 (0.57–0.69)
Model 1	Reference	0.93 (0.82–1.06)	0.93 (0.83–1.05)	0.73 (0.65–0.81)
Model 2	Reference	0.90 (0.74–1.08)	0.93 (0.82–1.05)	0.71 (0.59–0.84)
Model 3	Reference	0.96 (0.84–1.09)	1.00 (0.86–1.16)	0.79 (0.68–0.93)
Sensitivity analyses				
Without imputing				
N/n	607/11,248	418/7,374	475/7,828	1,224/25,798
Incidence rate, per 1,000 person-years	6.23 (5.75–6.75)	6.56 (5.96–7.22)	7.10 (6.49–7.77)	5.58 (5.27–5.90)
Multivariable-adjusted model	Reference	0.93 (0.82–1.05)	0.93 (0.82–1.05)	0.73 (0.65–0.82)
Excluding 566 participants who had events or death within 2 years of follow-up				
N/n	591/11,158	402/7,292	459/7,745	1,155/25,487
Incidence rate, per 1,000 person-years	6.07 (5.60–6.58)	6.32 (5.73–6.97)	6.87 (6.27–7.53)	5.27 (4.98–5.58)
Multivariable-adjusted model	Reference	0.92 (0.81–1.05)	0.93 (0.82–1.05)	0.72 (0.64, 0.80)
All-cause mortality				
N/n	538/11,248	423/7,374	489/7,828	1,786/25,798
Incidence rate, per 1,000 person-years	5.40 (4.96–5.88)	6.49 (5.90–7.13)	7.14 (6.53–7.80)	7.98 (7.61–8.35)
Unadjusted model	Reference	1.06 (0.93–1.20)	0.94 (0.83–1.06)	0.76 (0.68–0.83)
Model 1	Reference	1.05 (0.92–1.19)	0.94 (0.83–1.07)	0.89 (0.80–0.99)
Model 2	Reference	0.96 (0.80–1.15)	0.94 (0.83–1.06)	0.82 (0.70–0.96)
Model 3	Reference	1.05 (0.92–1.20)	0.95 (0.81–1.10)	0.89 (0.77–1.03)
Sensitivity analyses				
Without imputing				
N/n	538/11,248	423/7,374	489/7,828	1,786/25,798
Incidence rate, per 1,000 person-years	5.40 (4.96–5.88)	6.49 (5.90–7.13)	7.14 (6.53–7.80)	7.98 (7.61–8.35)
Model 1	Reference	1.05 (0.92–1.19)	0.93 (0.82–1.05)	0.89 (0.80–0.99)
Excluding 471 participants who had death within 2 years of follow-up				
N/n	462/11,172	352/7,303	419/7,758	1,532/25,544
Incidence rate, per 1,000 person-years	4.64 (4.24–5.08)	5.40 (4.87–6.00)	6.12 (5.56–6.74)	6.85 (6.51–7.20)
Model 1	Reference	1.02 (0.88–1.17)	0.93 (0.82–1.07)	0.86 (0.77–0.97)

*CVD, cardiovascular disease; HR, hazard ratio; CI, confidence interval. Model 1 Adjusted for sex, educational level (less than high school, high school/above), body mass index, estimated glomerular filtration rate (<30, 30 ≤eGFR <60, or ≥ 60 ml/min/1.73 m^2^), high-sensitivity C-reactive protein (<1.0, 1.0 ≤ Hs-CRP ≤ 3.0, or > 3.0 mg/L), low density lipoprotein cholesterol, high density lipoprotein cholesterol, hypertension, diabetes mellitus, and use of antihypertensive, hypoglycemic, and lipid-lowering medications (yes/no for each). Model 2 Included covariates in model 1 and health lifestyle score at 2006. Model 3 Included covariates in model 1 and health lifestyle score at 2010*.

After additional adjustments for 2006 or 2010 healthy lifestyle scores, the association remained with HRs of 0.71 (95% CI, 0.59–0.84) and 0.79 (95% CI, 0.68–0.93). Sensitivity analyses was conducted without imputing or excluding CVD events or death which occurred during the first 2 years of follow-up, the result remained stable.

Among 2,724 CVD events, 2,172 were strokes and 505 were myocardial infarction, 47 involved both (Supplementary Table 3). A decreased risk was also observed between healthy lifestyle score trajectories and stroke incidence, with HRs of 0.62 (95% CI, 0.55–0.69), 0.70 (95% CI, 0.62–0.79), 0.70 (95% CI, 0.58–0.84) and 0.77 (95% CI, 0.65–0.92) in unadjusted model and multivariable-adjusted model 1–3. Significantly associations with myocardial infarction were only observed in unadjusted model (HR, 0.67; 95% CI, 0.53–0.82).

Our analysis of all-cause mortality was provided in Table 2. 3,236 deaths were observed during the follow-up period. The incidence rate of low-stable, high-decreasing, low-increasing and high-stable trajectories were 5.40, 6.49, 7.14 and 7.98 per 1,000 person-years (PYs), respectively. Compared with low-stable trajectory, high-stable trajectory appeared to correlate with lower subsequent risk of all-cause mortality, with HRs of 0.76 (95% CI, 0.68–0.83) in unadjusted model and 0.89 (95% CI, 0.80–0.99) in the multivariable-adjusted model. These findings did not change after additional adjustment for the 2006 or 2010 healthy lifestyle scores, without imputing, or excluding CVD incidence which occurred during the first 2 years of follow-up. The HRs with corresponding confidence intervals were as follows: 0.82 (95% CI, 0.70–0.96), 0.89 (95% CI, 0.77–1.03), 0.89 (95% CI, 0.80–0.99) and 0.89 (95% CI, 0.80–0.99).

### Stratified Analyses

The results of stratified analyses according to sex and age in CVD events and all-cause mortality were present in Tables 3, 4. No significant interaction was observed between age, sex, and trajectories of healthy lifestyle scores (*p* > 0.050). There was however a negative relationship within the sexes, with high-stable trajectory and CVD incidence was observed in men (HR, 0.74; 95% CI, 0.67–0.83), and not in women (HR, 0.94; 95% CI, 0.13–6.74). Healthy lifestyle score trajectories were identified separately for men and women (Supplementary Figures 2, 3), and similar results were observed (Supplementary Tables 4, 5). A reduced risk of all-cause mortality appeared significant only in participants older than 60 years (HR, 0.82; 95% CI, 0.71–0.95).

**Table 3:** Stratified analysis of CVD and all-cause mortality according to sex.

	**Health lifestyle score trajectories, HR (95% CI)**				***P* for interaction**
	**Low-stable**	**High-decreasing**	**Low-increasing**	**High-stable**	
CVD					0.228
Male					
N/n	606/11,177	408/6,989	461/7,403	910/14,224	
Incidence rate, per 1,000 person-years	6.26 (5.78–6.78)	6.76 (6.13–7.45)	7.29 (6.66–7.99)	7.66 (7.18–8.18)	
Multivariable-adjusted model	Reference	0.93 (0.82–1.06)	0.93 (0.82–1.05)	0.74 (0.67–0.83)	
Female					
N/n	1/71	10/385	14/425	314/11,574	
Incidence rate, per 1,000 person-years	1.60 (0.23–11.34)	2.98 (1.60–5.54)	3.83 (2.27–6.47)	3.12 (2.79–3.48)	
Multivariable-adjusted model	Reference	1.36 (0.17–10.66)	2.18 (0.28–16.62)	0.94 (0.13–6.74)	
All-cause mortality					0.913
Male					
N/n	538/11,177	415/6,989	479/7,403	1,436/14,224	
Incidence rate, per 1,000 person-years	5.43 (4.99–5.91)	6.71 (6.10–7.39)	7.39 (6.76–8.08)	11.77 (11.17–12.39)	
Multivariable-adjusted model	Reference	1.06 (0.93–1.20)	0.95 (0.84–1.08)	0.91 (0.81–1.01)	
Female					
N/n	0/71	8/385	10/425	350/11,574	
Incidence rate, per 1,000 person-years	-	2.37 (1.18–4.73)	2.69 (1.45–4.99)	3.43 (3.09–3.81)	
Multivariable-adjusted model	Reference	-	-	-	

*HR, hazard ratio; CI, confidence interval. Adjusted for educational level (less than high school, high school/above), body mass index, estimated glomerular filtration rate (<30, 30 ≤ eGFR <60, or ≥ 60 ml/min/1.73 m^2^), high-sensitivity C-reactive protein (<1.0, 1.0 ≤ Hs-CRP ≤ 3.0, or > 3.0 mg/L), low density lipoprotein cholesterol, high density lipoprotein cholesterol, hypertension, diabetes mellitus, and use of antihypertensive, hypoglycemic, and lipid-lowering medications (yes/no for each)*.

**Table 4:** Stratified analysis of CVD and all-cause mortality according to age group.

	**Health lifestyle score trajectories, HR (95% CI)**				***P* for interaction**
	**Low-stable**	**High-decreasing**	**Low-increasing**	**High-stable**	
CVD					0.476
<60 y					
N/n	460/9,813	290/6,245	270/5,975	525/17,123	
Incidence rate, per 1,000 person-years	5.34 (4.87–5.85)	5.29 (4.71–5.93)	5.17 (4.59–5.82)	3.49 (3.21–3.81)	
Multivariable-adjusted model	Reference	0.90 (0.77–1.04)	0.92 (0.79–1.07)	0.77 (0.67–0.89)	
≥60 y					
N/n	147/1,435	128/1,129	205/1,853	699/8,675	
Incidence rate, per 1,000 person-years	13.03 (11.08–15.31)	14.44 (12.15–17.17)	14.05 (12.25–16.11)	10.10 (9.38–10.88)	
Multivariable-adjusted model	Reference	1.05 (0.83–1.33)	0.98 (0.79–1.21)	0.73 (0.61–0.88)	
All-cause mortality					0.201
<60 y					
N/n	314/9,813	242/6,245	212/5,975	430/17,123	
Incidence rate, per 1,000 person-years	3.57 (3.20–3.99)	4.33 (3.82–4.91)	3.98 (3.48–4.56)	2.82 (2.57–3.10)	
Multivariable-adjusted model	Reference	1.15 (0.97–1.36)	1.08 (0.91–1.29)	0.97 (0.83–1.15)	
≥60 y					
N/n	224/1,435	181/1129	277/1,853	1,356/8,675	
Incidence rate, per 1,000 person-years	18.98 (16.65–21.64)	19.37 (16.74–22.41)	18.07 (16.06–20.33)	18.93 (17.95–19.96)	
Multivariable-adjusted model	Reference	0.93 (0.76–1.13)	0.83 (0.69–0.99)	0.82 (0.71–0.95)	

*HR, hazard ratio; CI, confidence interval. Adjusted for sex, educational level (less than high school, high school/above), body mass index, estimated glomerular filtration rate (<30, 30 ≤ eGFR <60, or ≥ 60 ml/min/1.73 m^2^), high-sensitivity C-reactive protein (<1.0, 1.0 ≤ Hs-CRP ≤ 3.0, or > 3.0 mg/L), low density lipoprotein cholesterol, high density lipoprotein cholesterol, hypertension, diabetes mellitus, and use of antihypertensive, hypoglycemic, and lipid-lowering medications (yes/no for each)*.

Stratified analyses according to overweight/obesity, hypertension, diabetes and dyslipidemia were present in Supplementary Tables 6, 7. No significant interaction was observed between overweight/obesity, hypertension, diabetes and dyslipidemia and trajectories of healthy lifestyle scores either (*p* > 0.050). According to hypertension and diabetes status, reduced risk of CVD incidence was observed in those with hypertension (HR, 0.73; 95% CI, 0.64–0.82) and without diabetes (HR, 0.71; 95% CI, 0.62–0.80), and marginally in non-hypertensive participants (HR, 0.76; 95% CI, 0.59–1.00), but not in diabetes (HR, 0.83; 95% CI, 0.66–1.04). A reduced risk of all-cause mortality appeared marginally significant only in those without dyslipidemia (HR, 0.87; 95% CI, 0.76–1.00).

To gain deeper insight to the influence of BMI change included by many metrics, we performed a stratified analysis according to BMI change from 2006 to 2010: BMI decreased (<-1 kg/m^2^); BMI stable (−1–0.9 kg/m^2^); BMI increased (≥1 kg/m^2^), present in Table 5. Marginally significant interactions were observed between BMI change and trajectories of healthy lifestyle scores both on CVD and all-cause mortality, suggesting great difference among different BMI change level (*p* < 0.100). As for CVD, compared with low-stable trajectory, high-stable trajectory appears to negatively correlate with a lower subsequent risk of developing CVD in both BMI decreased (HR, 0.67; 95% CI, 0.55–0.82) and BMI stable group (HR, 0.72; 95% CI, 0.61–0.84). Surprisingly, low-increasing trajectory showed a reduced risk in BMI decreased (HR, 0.73; 95% CI, 0.57–0.93). As for all-cause mortality, apart from high-stable trajectory (HR, 0.83; 95% CI, 0.70–0.97), low-increasing trajectory (HR, 0.80; 95% CI, 0.66–0.96) showed reduced risk of death in BMI stable group. In addition, high-decreasing trajectory in BMI decreased group appeared higher risk of death (HR, 1.14; 95% CI, 1.13–1.83).

**Table 5:** Stratified analysis of CVD and all-cause mortality according to BMI change from 2006 to 2010.

	**Health lifestyle score trajectories, HR (95% CI)**				***P* for interaction**
	**Low-stable**	**High-decreasing**	**Low-increasing**	**High-stable**	
CVD					0.080
< −1 kg/m^2^ (*n* = 13,425)					
N/n	179/2,702	132/2,012	111/1,952	342/6,759	
Incidence rate, per 1,000 person-years	7.65 (6.61–8.85)	7.64 (6.44–9.06)	6.68 (5.55–8.05)	5.97 (5.37–6.64)	
Multivariable-adjusted model	Reference	0.95 (0.75–1.19)	0.73 (0.57–0.93)	0.67 (0.55–0.82)	
(−1 to 0.9) kg/m^2^ (*n* = 24,292)					
N/n	292/5,695	161/3,284	226/3,741	517/11,572	
Incidence rate, per 1,000 person-years	5.91 (5.27–6.63)	5.64 (4.83–6.58)	7.04 (6.18–8.02)	5.24 (4.80–5.71)	
Multivariable-adjusted model	Reference	0.86 (0.70–1.04)	0.99 (0.83–1.18)	0.72 (0.61–0.84)	
≥1 kg/m^2^ (*n* = 14,531)					
N/n	136/2,851	125/2,078	138/2,135	365/7,467	
Incidence rate, per 1,000 person-years	5.52 (4.67–6.53)	6.99 (5.86–8.33)	7.60 (6.44–8.99)	5.75 (5.19–6.37)	
Multivariable-adjusted model	Reference	1.02 (0.80–1.31)	1.08 (0.85–1.37)	0.83 (0.67–1.04)	
All-cause mortality					0.057
< −1 kg/m^2^ (*n* = 13,425)					
N/n	128/2,702	143/2,012	152/1,952	550/6,759	
Incidence rate, per 1,000 person-years	5.32 (4.47–6.32)	8.05 (6.83–9.48)	8.94 (7.62–10.48)	9.39 (8.64–10.21)	
Multivariable-adjusted model	Reference	1.44 (1.13–1.83)	1.22 (0.96–1.55)	1.05 (0.85–1.30)	
(−1 to 0.9) kg/m^2^ (*n* = 24,292)					
N/n	268/5,695	160/3,284	196/3,741	727/11,572	
Incidence rate, per 1,000 PYS	5.31 (4.71–5.99)	5.49 (4.70–6.41)	5.96 (5.18–6.85)	7.23 (6.72–7.77)	
Multivariable-adjusted model	Reference	0.90 (0.74–1.10)	0.80 (0.66–0.96)	0.83 (0.70–0.97)	
≥1 kg/m^2^ (*n* = 14,531)					
N/n	142/2,851	120/2,078	141/2,135	509/7,467	
Incidence rate, per 1,000 person-years	5.66 (4.80–6.67)	6.56 (5.48–7.84)	7.57 (6.42–8.93)	7.86 (7.20–8.57)	
Multivariable-adjusted model	Reference	0.94 (0.73–1.20)	0.94 (0.74–1.19)	0.83 (0.68–1.02)	

*HR, hazard ratio; CI, confidence interval. Adjusted for sex, educational level (less than high school, high school/above), estimated glomerular filtration rate (<30, 30 ≤ eGFR <60, or ≥60 ml/min/1.73 m^2^), high-sensitivity C-reactive protein (<1.0, 1.0 ≤ Hs-CRP ≤ 3.0, or >3.0 mg/L), low density lipoprotein cholesterol, high density lipoprotein cholesterol, hypertension, diabetes mellitus, and use of antihypertensive, hypoglycemic, and lipid-lowering medications (yes/no for each)*.

## Discussion

In this prospective study, four distinct healthy lifestyle trajectories were identified and maintaining a healthy lifestyle over 6 years was associated with a 27% lower risk of CVD incidence and an 11% lower risk of all-cause mortality, when compared with a consistently unhealthy lifestyle. Such correlations are seldom influenced by demographics or disease history, though reduced risk of encountering CVD events was observed only in men and in those without diabetes, while a reduced risk of all-cause mortality was only observed in those older than 60 years and without dyslipidemia. However, such correlations are quite different when considering BMI change. Maintaining a healthy lifestyle appeared to negatively correlate with a lower subsequent risk of developing CVD when BMI decreased or stable and all-cause mortality when BMI was stable. Changing lifestyle to a healthy way was showed a reduced risk of cardiovascular disease only when BMI decreased and all-cause mortality only when BMI was stable.

A previous study based on the same cohort found a protective impact in maintaining good cardiovascular health defined according to the American Heart Association (AHA) guidelines for ischemic and intracerebral hemorrhagic stroke (14). However, these healthy lifestyle patterns have two components i.e., lifestyle and physiological measures, such as BMI. These data could be manifestations of specific medicines or lifestyles, and therefore these combined metrics could confound the effect of lifestyle. Therefore, we focused on lifestyle trajectories and our findings are consistent with previous studies, suggesting long-term maintenance of a healthy lifestyle is associated with decreased CVD risk (HR, 0.73; 95% CI, 0.65–0.81) and all-cause mortality (HR, 0.89; 95% CI, 0.80–0.99). Considering BMI, we found baseline BMI level did not change such a negative relationship. However, when further considering the influence of BMI change, we observed a negative association between a healthy lifestyle and reduced risk of CVD in those with reduced (HR, 0.67; 95% CI, 0.55–0.82) or stable BMI (HR, 0.72; 95% CI, 0.61–0.84). The causal relationship between increased adiposity and the risk of incident CVD events was proved by observational and genetics-driven studies (22). Similarly, we also observed that the high-stable trajectory was associated with lower risk of all-cause mortality in those with stable BMI (HR, 0.83; 95% CI, 0.70–0.97), compared with low-stable trajectory, These results highlighted the importance of the maintenance of BMI in preventing early death, as pronounced weight change, not only weight gain, is associated with an increase in all-cause mortality risk (23, 24).

A shift toward a healthier lifestyle during early life will decrease long-term risk of CVD has been reported by many short- or long-term, prospective interventional studies. For example, we know that those in engaging in a healthy lifestyle intervention is associated with a lower level of risk and therefore a reduction in predicted CVD risk (25–27). However, lifestyle changes which occur across the life course, with the application of interventions are becoming increasingly less likely. Further qualitative research focusing on dynamics of lifestyles and development/aging is needed. Although, two prospective cohorts reported having observed improved lifestyles in the short term could reduce risk of CVD or death, but both studies included BMI (28, 29). However, we did not observe such benefits when focused on lifestyles only, as we did not observe any association between CVD or all-cause mortality risk and the low-increasing trajectory. Similarly, a study in the UK reported a decrease in unhealthy lifestyle scores was not associated with a beneficial effect on mortality (HR, 0.93; 95% CI, 0.83–1.04), either (30). When we further divided participants according to BMI change, a negative relationship between the low-increasing trajectory and CVD incidence was observed in the decreased BMI group (HR, 0.73; 95% CI, 0.57–0.93), and in deaths in the stable BMI group (HR, 0.80; 95% CI, 0.66–0.96). This suggests that the protective effect of improved lifestyles may be gained only when BMI is reduced, which is consistent with previous research (31). However, maintaining BMI, even with a lifestyle changes, is important due to a “U” shaped relationship between BMI and all-cause mortality (24).

After sex, age, overweight/obesity, hypertension, diabetes and dyslipidemia were stratified, we observed a reduced risk in CVD in men. It is acknowledged that men are more likely to have unhealthy lifestyles like smoking and drinking (32), which was also can be observed in our trajectories in men and women, so the benefit would be more obvious in men who maintaining a healthy lifestyle. In another way, women sample in our cohort is relatively limited leading to a small number of outcomes, so it may be hard to see the association. All patients could benefit from healthy lifestyles, but different from the overweight/obesity and hypertension, reduced risk in CVD was only observed in those without diabetes in our study. Maybe for diabetes patients, changing/maintenance of healthy lifestyles was not enough, intensive intervention for glucose control was needed. As for all-cause mortality, we observed reduced risk in those older than 60 years, as they are facing greater death threat and are likely to expose to unhealthy lifestyles for a longer time. A decreased risk was observed between the high-stable trajectory and stroke incidence with an HR of 0.70 (95% CI, 0.62–0.79), but not myocardial infarction (HR, 0.82; 95% CI, 0.64–1.04) after adjusting potential confounders. In our cohort, stroke accounted for about 80% of CVD, so the myocardial infarction incidence was relatively low. This insignificant phenomenon on myocardial infarction was prevalent in Kailuan cohort studies (33, 34). Also, myocardial infarction was all ischemic, but stroke was both ischemic and hemorrhagic. So, the effect may be different depending on whether CVD is either ischemic and hemorrhagic. In this instance, we did not gather detailed information around further classifications and therefore we were unable to analyse this further.

Foundational research has shown that Chinese lifestyles are quite different from global communities. Kim et al. found lifestyles were slightly healthier in China than in the US, especially in terms of diet quality, physical activity, and smoking (35). However, this appears controversial because the Report on Nutrition and Chronic Disease Status of Chinese Residents suggested that unhealthy lifestyles remain prevalent (36). Therefore, it is safe to assume that lifestyles across Chinese populations are incredibly diverse. Zhu et al. found that compared to the differences between urban and rural areas, the differences between project areas are more profound. For example, in Henan, which is a neighboring province to Tangshan, Hebei Province, had relatively healthier lifestyles (32). Therefore, caution must be taken because trajectories can (at best) only represent Hebei region, rather than every region, or indeed China in general.

On that note, before making any recommendations it is important to first discuss the limitations involved. Firstly, our cohort only included Chinese adults from the Kailuan community. As has been mentioned, findings may not be generalizable to other populations but this does provide an opportunity to make both national and international comparisons. Similar associations between individuals and a number of healthy lifestyle metrics and CVD/mortality risks have been shown in multiple racial/ethnic groups, highlighting the broad generalizable nature of the data (28–30). Another limitation is the substitution of daily salt intake for diet quality as detailed dietary components data were not available until 2014 in our cohort. Given that salt intake was consistently found to be associated with higher CVD risk (37) and excessive salt intake is a problem in China, salt intake was used as a surrogate for diet quality.

## Conclusion

In this prospective study, four distinct healthy lifestyle score trajectories were identified. Maintaining a healthy lifestyle over 6 years was associated with a 27% lower risk of incident CVD and an 11% lower risk of all-cause mortality, compared with those with a consistently unhealthy lifestyle. Improving lifestyle is likely to reduce risk of CVD when BMI also decreases although the risk of all-cause mortality reduces when BMI remains stable. Our findings highlight the importance of strong public health efforts to improve lifestyle for CVD prevention.

## Data Availability Statement

The original contributions presented in the study are included in the article/Supplementary Material, further inquiries can be directed to the corresponding authors.

## Ethics Statement

The studies involving human participants were reviewed and approved by the Ethics Committee of the Kailuan General Hospital. The patients/participants provided their written informed consent to participate in this study.

## Author Contributions

SW, XiaodY, and YL: designed the study, reviewed and revised the manuscript. XD, WF, and XiaojY: coded and analyzed the data. XD, WF, XY, and SS: wrote the manuscript. SC and GW: collected data. YW and HZ: helped interpret the data. All authors read and approved the final manuscript.

## Funding

This study was supported by the Natural Science Foundation of Hebei Province (H2021209018) and Tangshan Science and Technology Innovation Team Program (20130206D).

## Conflict of Interest

The authors declare that the research was conducted in the absence of any commercial or financial relationships that could be construed as a potential conflict of interest.

## Publisher's Note

All claims expressed in this article are solely those of the authors and do not necessarily represent those of their affiliated organizations, or those of the publisher, the editors and the reviewers. Any product that may be evaluated in this article, or claim that may be made by its manufacturer, is not guaranteed or endorsed by the publisher.
