# Supplementary material for: Associations Between Healthy Lifestyle Trajectories and the Incidence of Cardiovascular Disease With All-Cause Mortality: A Large, Prospective, Chinese Cohort Study
Source: Front Cardiovasc Med. 2021 Dec 20;8:790497. doi: 10.3389/fcvm.2021.790497 (PMC8720765; doi:10.3389/fcvm.2021.790497)
Supplement: Supplementary file 1 [file Data_Sheet_1.docx]

**Supplementary materials**

**Contents**

**Supplementary Figure 1**. Flow chart for participant selection

**Supplementary Figure 2.** Mean healthy lifestyle scores in 2006, 2008 and 2010, according to four healthy lifestyle score trajectories in men

**Supplementary Figure 3.** Mean healthy lifestyle score in 2006, 2008 and 2010, according to four healthy lifestyle score trajectories in women

**Supplementary Table 1.** Definitions of Poor (0 Point), Intermediate (1 Point), and Ideal (2 Points) for each component of healthy lifestyle scores

**Supplementary Table 2.** Missing covariate data

**Supplementary Table 3.** Incidence of stroke and myocardial infarction according to healthy lifestyle score trajectories from 2006 to 2010

**Supplementary Table 4.** Incidence of CVD and all-cause mortality according to healthy lifestyle score trajectories from 2006 to 2010 in men

**Supplementary Table 5.** Incidence of CVD and all-cause mortality according to healthy lifestyle score trajectories from 2006 to 2010 in women

**Supplementary Table 6.** Stratified analysis of CVD according to disease history

**Supplementary Table 7.** Stratified analysis of all-cause mortality according to disease history


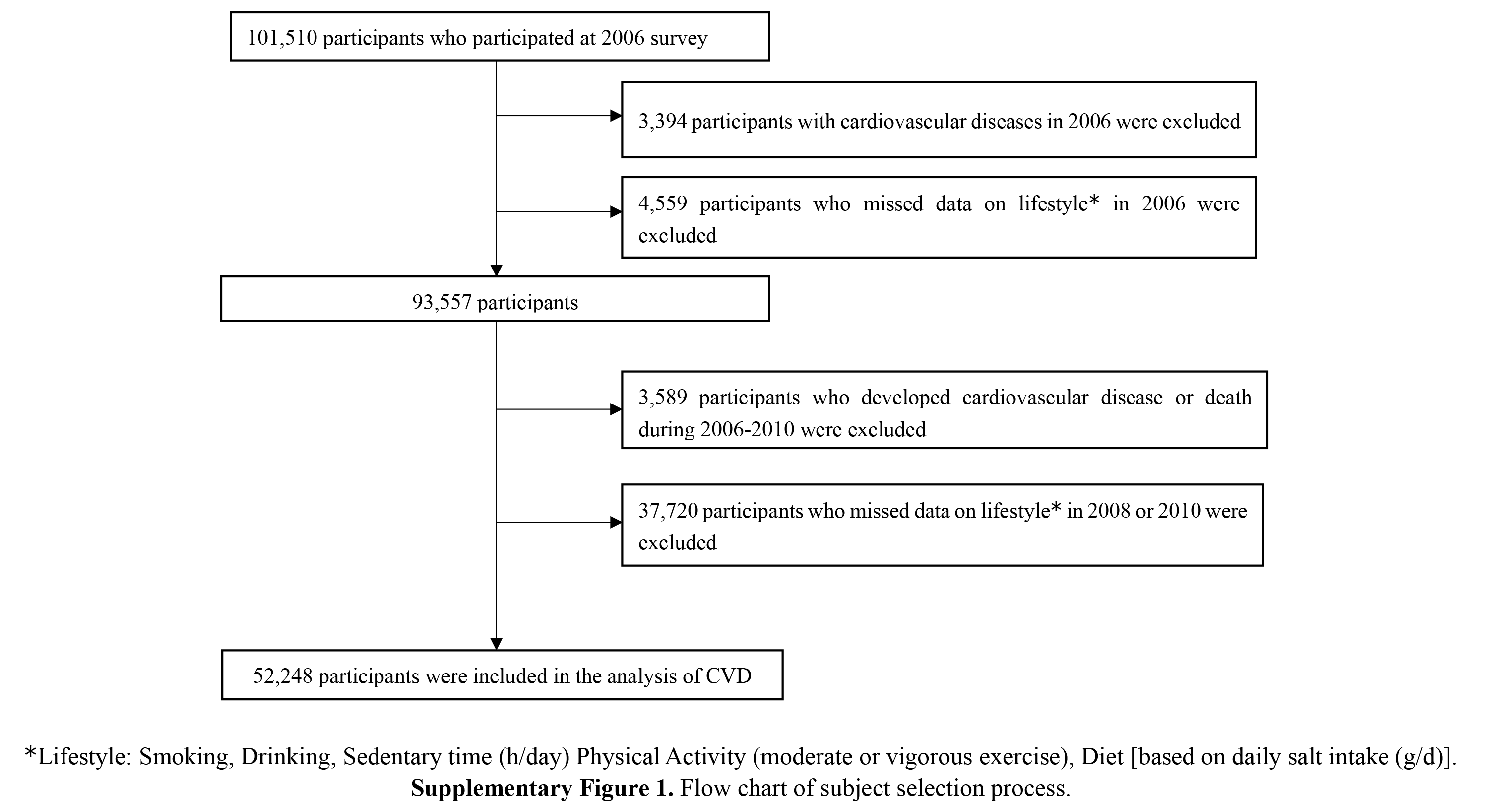


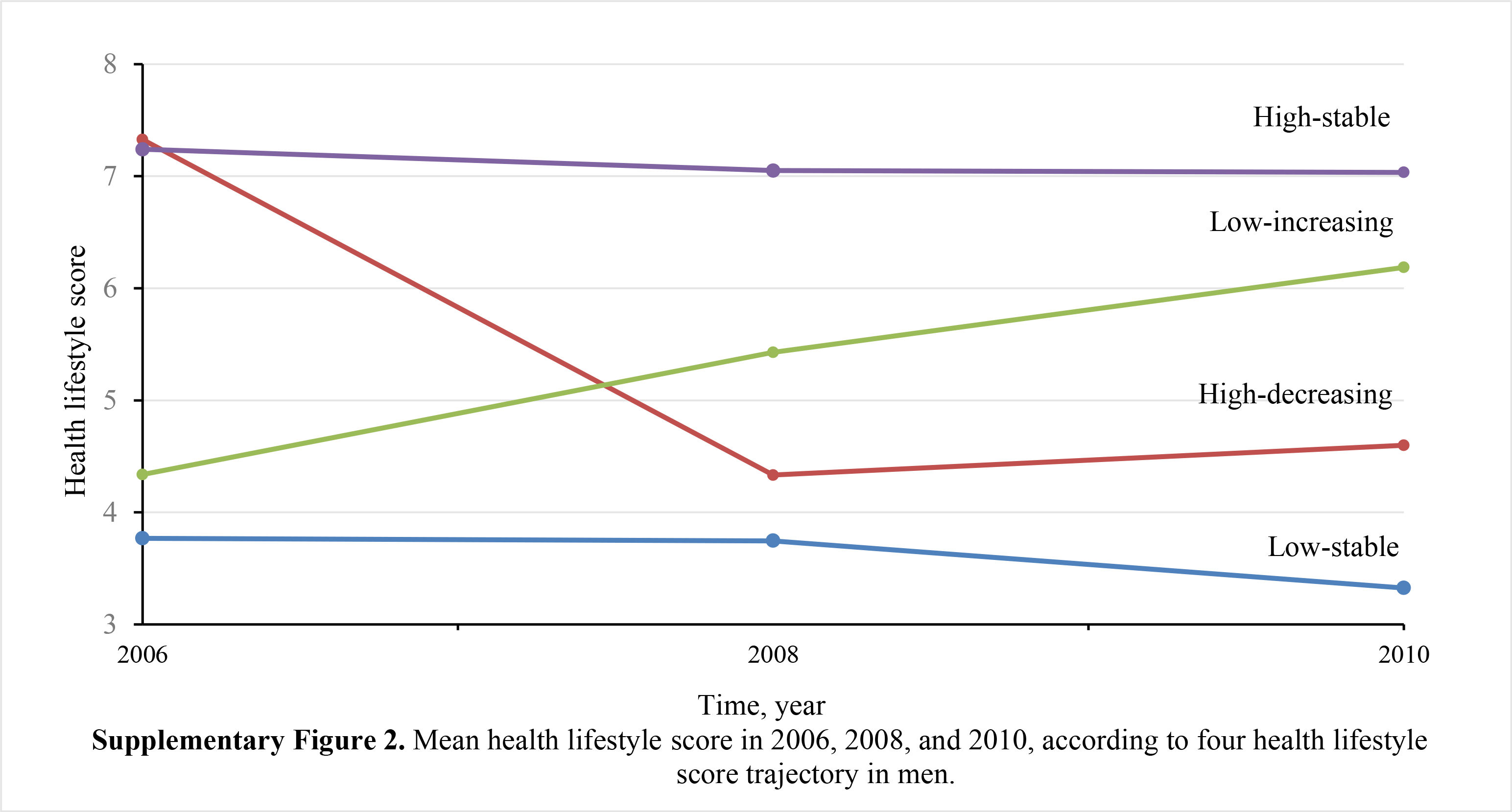


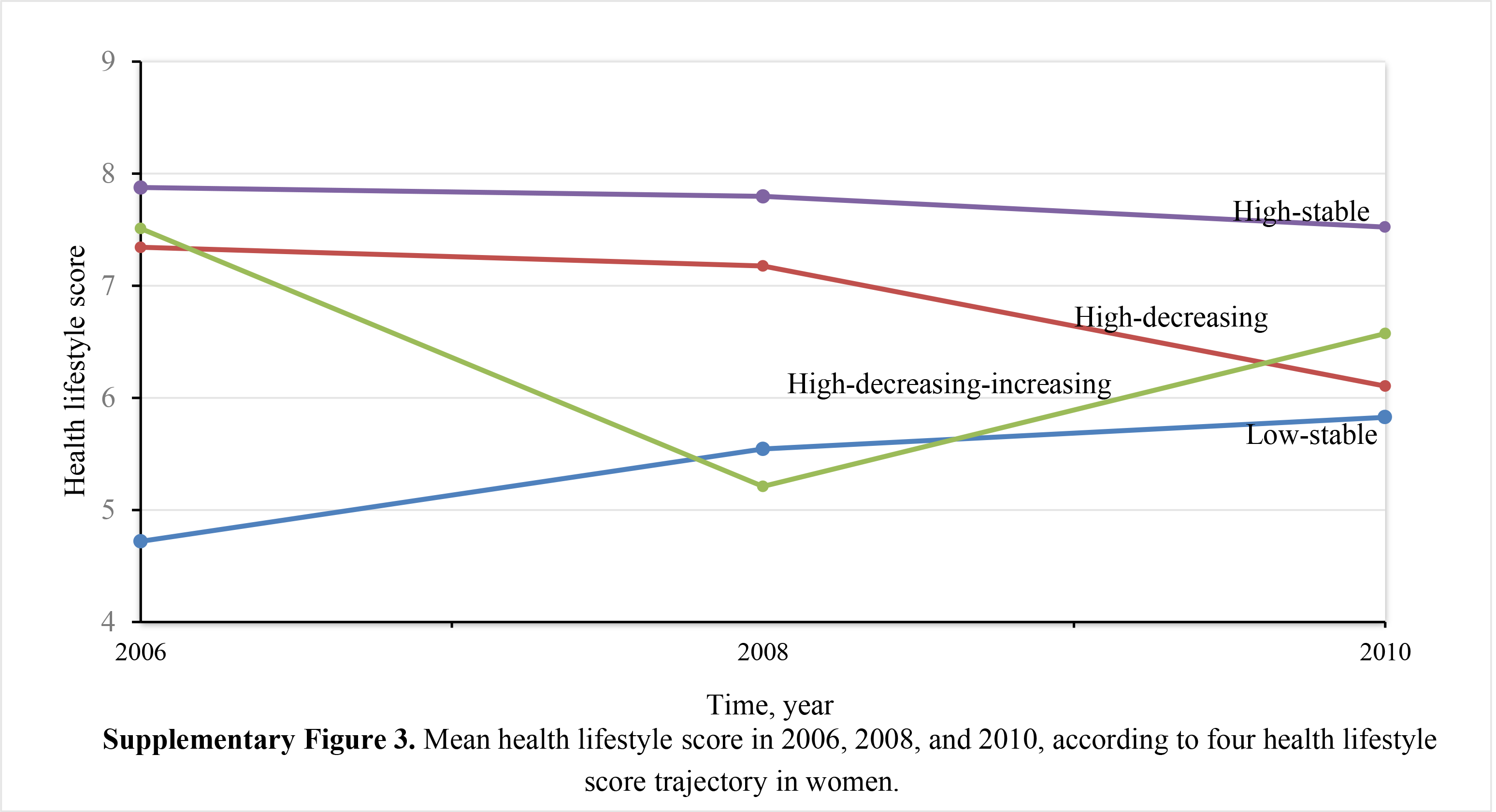


**Supplementary Table S1.** Definitions of Poor (0 Point), Intermediate (1 Point), and Ideal (2 Points) for Each Component of Healthy Lifestyle Score

| **Healthy Lifestyle Score Component** | **Poor (0 Point)** | **Intermediate (1 Point)** | **Ideal (2 Points)** |
| --- | --- | --- | --- |
| Cigarette Smoking | Current smoker | Past smoker^*^ | Never |
| Cigarette Drinking | Current drinker | Past drinker^*^ | Never |
| Sedentary time, h/day | ≥8 | 4-7 | <4 |
| Physical Activity (moderate or vigorous exercise) | No physical activity | physical activity (20+ minutes per  time) 1-2 times per week, during leisure time | physical activity (20+ minutes per time) 3 times per week, during leisure time |
| Diet, based on daily salt intake (g/d) | ≥10 | 6-9 | <6 |

^*^Past smoker/drinker was defined when a participant who reported to smoke/drink previously but quit smoking/drinking during the survey

**Supplementary Table 2.** Missing covariate data

| **Covariates** | **Total** | **Missing (%)** | **Imputed** | **Used for imputation** |
| --- | --- | --- | --- | --- |
| Age | 52,248 | 0 |  |  |
| Sex | 52,248 | 0 |  |  |
| Educational background | 52,248 | 0 |  |  |
| BMI | 52,248 | 3 (0.01) |  |  |
| SBP | 52,248 | 93 (0.18) |  |  |
| DBP | 52,248 | 84 (0.16) |  |  |
| FBG | 52,248 | 68 (0.13) |  |  |
| Hs-CRP | 52,248 | 99 (0.19) |  |  |
| eGFR | 52,248 | 95 (0.18) |  |  |
| LDL-C | 52,248 | 96 (0.18) |  |  |
| HDL-C | 52,248 | 95 (0.18) |  |  |
| Hypertension | 52,248 | 0 |  |  |
| Diabetes mellitus | 52,248 | 0 |  |  |
| Using of antihypertensive agent | 52,248 | 0 |  |  |
| Use of hypoglycemic medication | 52,248 | 0 |  |  |
| Use of lipid-lowering medication | 52,248 | 0 |  |  |

Abbreviations: BMI, body mass index; SBP, systolic blood pressure; DBP, diastolic blood pressure; FBG, fasting blood glucose; Hs-CRP, hypersensitive C-reactive protein; eGFR, estimated glomerular filtration rate; LDL-C, Low-density lipoprotein cholesterol; HDL, High-density lipoprotein cholesterol

**Supplementary Table 3.** Incidence of stroke and myocardial infarction according to healthy lifestyle score trajectories from 2006 to 2010

|  | **Health lifestyle score trajectories, HR (95% CI)** | | | |
| --- | --- | --- | --- | --- |
|  | **Low-stable** | **High-decreasing** | **Low-increasing** | **High-stable** |
| Stroke |  |  |  |  |
| N/n | 498/11,248 | 341/7,374 | 390/7,828 | 990/25,798 |
| Incidence rate, per 1000 PYs | 5.09 (4.66-5.55) | 5.32 (4.79-5.92) | 5.81 (5.26-6.41) | 4.49 (4.22-4.78) |
| Unadjusted model | Reference | 0.96 (0.84-1.11) | 0.94 (0.83-1.08) | 0.62 (0.55-0.69) |
| Model 1 | Reference | 0.92 (0.80-1.06) | 0.94 (0.82-1.07) | 0.70 (0.62-0.79) |
| Model 2 | Reference | 0.91 (0.74-1.12) | 0.93 (0.82-1.07) | 0.70 (0.58-0.84) |
| Model 3 | Reference | 0.95 (0.82-1.10) | 1.01 (0.85-1.19) | 0.77 (0.65-0.91) |
| Myocardial infarction |  |  |  |  |
| N/n | 122/11,248 | 83/7,374 | 91/7,828 | 256/25,798 |
| Incidence rate, per 1000 PYs | 1.23 (1.03-1.47) | 1.28 (1.03-1.59) | 1.33 (1.09-1.64) | 1.15 (1.02-1.30) |
| Unadjusted model | Reference | 0.96 (0.73-1.27) | 0.89 (0.68-1.18) | 0.66 (0.53-0.82) |
| Model 1 | Reference | 0.90 (0.68-1.20) | 0.87 (0.66-1.15) | 0.82 (0.64-1.04) |
| Model 2 | Reference | 0.87 (0.57-1.32) | 0.87 (0.66-1.14) | 0.79 (0.54-1.15) |
| Model 3 | Reference | 0.92 (0.68-1.23) | 0.91 (0.65-1.27) | 0.86 (0.62-1.21) |

Abbreviations: HR, hazard ratio; CI, confidence interval; PYs, person-years.

Model 1 Adjusted for sex, educational level (less than high school, high school/above), body mass index, estimated glomerular filtration rate (<30, 30 ≤eGFR<60, or ≥ 60 ml/min/1.73 m^2^), high-sensitivity C-reactive protein (< 1.0, 1.0 ≤ Hs-CRP ≤ 3.0, or > 3.0 mg/L), low density lipoprotein cholesterol, high density lipoprotein cholesterol, hypertension, diabetes mellitus, and use of antihypertensive, hypoglycemic, and lipid-lowering medications (yes/no for each).

Model 2 Included covariates in model 1, and health lifestyle score at 2006.

Model 3 Included covariates in model 1, and health lifestyle score at 2010.

**Supplementary Table 4.** Incidence of CVD and all-cause mortality according to healthy lifestyle score trajectories from 2006 to 2010 in men

|  | **Health lifestyle score trajectories, HR (95% CI)** | | | |
| --- | --- | --- | --- | --- |
|  | **Low-stable** | **High-decreasing** | **Low-increasing** | **High-stable** |
| CVD |  |  |  |  |
| N/n | 640/11,798 | 411/7,097 | 417/6,588 | 917/14,310 |
| Incidence rate, per 1000 PYs | 6.26 (5.79-6.76) | 6.69 (6.08-7.37) | 7.43 (6.75-8.18) | 7.68 (7.20-8.20) |
| Unadjusted model | Reference | 0.98 (0.87-1.11) | 0.98 (0.86-1.11) | 0.80 (0.72-0.89) |
| Model 1 | Reference | 0.92 (0.81-1.05) | 0.93 (0.82-1.05) | 0.75 (0.67-0.84) |
| Model 2 | Reference | 0.90 (0.74-1.09) | 0.93 (0.82-1.05) | 0.73 (0.61-0.87) |
| Model 3 | Reference | 0.95 (0.84-1.09) | 1.01 (0.86-1.18) | 0.83 (0.70-0.97) |
| All-cause mortality |  |  |  |  |
| N/n | 556/11,798 | 426/7,097 | 446/6,588 | 1,440/14,310 |
| Incidence rate, per 1000 PYs | 5.32 (4.89-5.78) | 6.78 (6.16-7.45) | 7.75 (7.06-8.51) | 11.73 (11.14-12.36) |
| Unadjusted model | Reference | 1.12 (0.99-1.28) | 1.04 (0.91-1.18) | 0.98 (0.89-1.09) |
| Model 1 | Reference | 1.09 (0.96-1.24) | 1.01 (0.89-1.14) | 0.94 (0.84-1.04) |
| Model 2 | Reference | 1.01 (0.84-1.20) | 1.00 (0.88-1.14) | 0.87 (0.74-1.02) |
| Model 3 | Reference | 1.10 (0.96-1.25) | 1.02 (0.87-1.19) | 0.95 (0.81-1.10) |

Abbreviations: CVD, cardiovascular disease; HR, hazard ratio; CI, confidence interval; PYs, person-years.

Model 1 Adjusted for educational level (less than high school, high school/above), body mass index, estimated glomerular filtration rate (<30, 30 ≤eGFR<60, or ≥ 60 ml/min/1.73 m^2^), high-sensitivity C-reactive protein (< 1.0, 1.0 ≤ Hs-CRP ≤ 3.0, or > 3.0 mg/L), low density lipoprotein cholesterol, high density lipoprotein cholesterol, hypertension, diabetes mellitus, and use of antihypertensive, hypoglycemic, and lipid-lowering medications (yes/no for each).

Model 2 included covariates in model 1, and health lifestyle score at 2006.

Model 3 included covariates in model 1, and health lifestyle score at 2010.

**Supplementary Table 5.** Incidence of CVD and all-cause mortality according to healthy lifestyle score trajectories from 2006 to 2010 in women

|  | **Health lifestyle score trajectories, HR (95% CI)** | | | |
| --- | --- | --- | --- | --- |
|  | **Low-stable** | **High-decreasing** | **High-decreasing -increasing** | **High-stable** |
| **CVD** |  |  |  |  |
| N/n | 13/499 | 38/2,087 | 15/583 | 273/9,286 |
| Incidence rate, per 1000 PYs | 3.02 (1.75-5.20) | 2.09 (1.52-2.87) | 2.93 (1.77-4.86) | 3.38 (3.00-3.81) |
| Unadjusted model | Reference | 0.56 (0.30-1.05) | 0.86 (0.41-1.82) | 0.66 (0.38-1.16) |
| Model 1 | Reference | 0.53 (0.28-1.00) | 0.72 (0.34-1.53) | 0.56 (0.32-0.99) |
| Model 2 | Reference | 0.54 (0.26-1.10) | 0.73 (0.31-1.70) | 0.57 (0.28-1.16) |
| Model 3 | Reference | 0.53 (0.28-1.00) | 0.73 (0.34-1.54) | 0.57 (0.31-1.03) |
| **All-cause mortality** |  |  |  |  |
| N/n | 10/499 | 52/2,087 | 11/583 | 295/9,286 |
| Incidence rate, per 1000 PYs | 2.28 (1.23-4.24) | 2.83 (2.16-3.72) | 2.13 (1.18-3.85) | 3.61 (3.22-4.04) |
| Unadjusted model | Reference | 1.00 (0.51-1.96) | 0.87 (0.37-2.05) | 0.89 (0.47-1.68) |
| Model 1 | Reference | 0.95 (0.48-1.88) | 0.78 (0.33-1.84) | 0.77 (0.41-1.46) |
| Model 2 | Reference | 0.92 (0.43-1.96) | 0.74 (0.29-1.91) | 0.73 (0.34-1.58) |
| Model 3 | Reference | 0.95 (0.48-1.87) | 0.79 (0.33-1.87) | 0.79 (0.41-1.52) |

Abbreviations: CVD, cardiovascular disease; HR, hazard ratio; CI, confidence interval; PYs, person-years.

Model 1 Adjusted for educational level (less than high school, high school/above), body mass index, estimated glomerular filtration rate (<30, 30 ≤eGFR<60, or ≥ 60 ml/min/1.73 m^2^), high-sensitivity C-reactive protein (< 1.0, 1.0 ≤ Hs-CRP ≤ 3.0, or > 3.0 mg/L), low density lipoprotein cholesterol, high density lipoprotein cholesterol, hypertension, diabetes mellitus, and use of antihypertensive, hypoglycemic, and lipid-lowering medications (yes/no for each).

Model 2 included covariates in model 1, and health lifestyle score at 2006.

Model 3 included covariates in model 1, and health lifestyle score at 2010.

**Supplementary Table 6.** Stratified analysis of CVD according to disease history

|  | **Health lifestyle score trajectories, HR (95% CI)** | | | | **P for interaction** | |
| --- | --- | --- | --- | --- | --- | --- |
|  | **Low-stable** | **High-decreasing** | **Low-increasing** | **High-stable** |  |  |
| **Overweight/obesity** |  |  |  |  | | 0.737 |
| BMI <24 kg/m^2^ |  |  |  |  | |  |
| N/n | 209/4,320 | 125/2,733 | 139/2,925 | 355/10,252 | |  |
| Incidence rate, per 1000 PYs | 5.57 (4.86-6.37) | 5.26 (4.41-6.26) | 5.55 (4.70-6.55) | 4.05 (3.65-4.49) | |  |
| Multivariable-adjusted model | Reference | 0.82 (0.65-1.02) | 0.80 (0.64-1.00) | 0.59 (0.49-0.72) | |  |
| BMI ≥24 kg/m^2^ |  |  |  |  | |  |
| N/n | 398/6,928 | 293/4,641 | 336/4,903 | 869/15,546 | |  |
| Incidence rate, per 1000 PYs | 6.65 (6.02-7.33) | 7.34 (6.54-8.23) | 8.04 (7.22-8.94) | 6.59 (6.17-7.04) | |  |
| Multivariable-adjusted model | Reference | 0.98 (0.84-1.14) | 1.00 (0.86-1.15) | 0.80 (0.70-0.92) | |  |
| **Hypertension** |  |  |  |  | | 0.762 |
| No |  |  |  |  | |  |
| N/n | 111/4,870 | 76/2,935 | 90/3,387 | 200/11,445 | |  |
| Incidence rate, per 1000 PYs | 2.57 (2.14-3.10) | 2.93 (2.34-3.66) | 3.02 (2.46-3.72) | 2.00 (1.74-2.29) | |  |
| Multivariable-adjusted model | Reference | 0.94 (0.70-1.27) | 1.04 (0.78-1.37) | 0.76 (0.59-1.00) | |  |
| Yes |  |  |  |  | |  |
| N/n | 496/6,378 | 342/4,439 | 385/4,441 | 124/14,353 | |  |
| Incidence rate, per 1000 PYs | 9.14 (8.37-9.98) | 9.06 (8.15-10.07) | 10.38 (9.39-11.47) | 8.59 (8.08-9.13) | |  |
| Multivariable-adjusted model | Reference | 0.92 (0.80-1.06) | 0.91 (0.80-1.05) | 0.73 (0.64-0.82) | |  |
| **Diabetes mellitus** |  |  |  |  | | 0.455 |
| No |  |  |  |  | |  |
| N/n | 482/9,962 | 321/6,437 | 343/6,802 | 891/22,289 | |  |
| Incidence rate, per 1000 PYs | 5.56 (5.08-6.08) | 5.74 (5.14-6.40) | 5.86 (5.27-6.51) | 4.66 (4.36-4.97) | |  |
| Multivariable-adjusted model | Reference | 0.92 (0.80-1.06) | 0.87 (0.76-1.01) | 0.71 (0.62-0.80) | |  |
| Yes |  |  |  |  | |  |
| N/n | 125/1,286 | 97/937 | 132/1,026 | 333/3,509 | |  |
| Incidence rate, per 1000 PYs | 11.68 (9.80-13.91) | 12.51 (10.25-15.27) | 15.87 (13.38-18.82) | 11.86 (10.65-13.20) | |  |
| Multivariable-adjusted model | Reference | 0.97 (0.74-1.27) | 1.15 (0.89-1.47) | 0.83 (0.66-1.04) | |  |
| **Dyslipidemia** |  |  |  |  | | 0.513 |
| No |  |  |  |  | |  |
| N/n | 287/6,603 | 236/4,707 | 232/4,768 | 672/17,306 | |  |
| Incidence rate, per 1000 PYs | 4.98 (4.43-5.59) | 5.77 (5.08-6.56) | 5.65 (4.97-6.43) | 4.53 (4.20-4.88) | |  |
| Multivariable-adjusted model | Reference | 1.01 (0.85-1.20) | 0.90 (0.75-1.07) | 0.72 (0.62-0.84) | |  |
| Yes |  |  |  |  | |  |
| N/n | 320/4,645 | 182/2,667 | 243/3,060 | 552/8,492 | |  |
| Incidence rate, per 1000 PYs | 8.05 (7.21-8.98) | 7.97 (6.89-9.22) | 9.41 (8.30-10.67) | 7.77 (7.15-8.45) | |  |
| Multivariable-adjusted model | Reference | 0.87 (0.73-1.05) | 0.98 (0.83-0.16) | 0.77 (0.66-0.90) | |  |

Abbreviations: HR, hazard ratio; CI, confidence interval; BMI, body mass index; PYs, person-years.

Adjusted for sex, educational level (less than high school, high school/above), body mass index, estimated glomerular filtration rate (<30, 30 ≤eGFR<60, or ≥ 60 ml/min/1.73 m^2^), high-sensitivity C-reactive protein (< 1.0, 1.0 ≤ Hs-CRP ≤ 3.0, or > 3.0 mg/L), low density lipoprotein cholesterol, high density lipoprotein cholesterol, hypertension, diabetes mellitus, and use of antihypertensive, hypoglycemic, and lipid-lowering medications (yes/no for each).

**Supplementary Table 7.** Stratified analysis of all-cause mortality according to disease history

|  | **Health lifestyle score trajectories, HR (95% CI)** | | | | **P for interaction** | |
| --- | --- | --- | --- | --- | --- | --- |
|  | **Low-stable** | **High-decreasing** | **Low-increasing** | **High-stable** |  |  |
| **Overweight/obesity** |  |  |  |  | | 0.314 |
| BMI <24 kg/m^2^ |  |  |  |  | |  |
| N/n | 232/4,320 | 151/2,733 | 190/2,925 | 692/10,252 | |  |
| Incidence rate, per 1000 PYs | 6.07 (5.33-6.90) | 6.24 (5.32-7.32) | 7.44 (6.45,8.57) | 7.78 (7.22,8.38) | |  |
| Multivariable-adjusted model | Reference | 0.93 (0.75-1.14) | 0.96 (0.79-1.17) | 0.88 (0.74-1.04) | |  |
| BMI ≥24 kg/m^2^ |  |  |  |  | |  |
| N/n | 306/6,928 | 272/4,641 | 299/4,903 | 1,904/15,546 | |  |
| Incidence rate, per 1000 PYs | 4.98 (4.46-5.58) | 6.63 (5.89-7.46) | 6.96 (6.21-7.79) | 8.10 (7.64-8.60) | |  |
| Multivariable-adjusted model | Reference | 1.13 (0.96-1.33) | 0.94(0.80-1.11) | 0.90 (0.78-1.03) | |  |
| **Hypertension** |  |  |  |  | | 0.210 |
| No |  |  |  |  | |  |
| N/n | 143/4,870 | 94/2,935 | 104/3,387 | 388/11,445 | |  |
| Incidence rate, per 1000 PYs | 3.29 (2.79-3.87) | 3.58 (2.93-4.39) | 3.46 (2.85-4.19) | 3.84 (3.48-4.25) | |  |
| Multivariable-adjusted model^※^ | Reference | 0.91 (0.70-1.19) | 0.87 (0.67-1.12) | 0.89 (0.72-1.11) | |  |
| Yes |  |  |  |  | |  |
| N/n | 395/6,378 | 329/4,439 | 385/4,441 | 1,398/14,353 | |  |
| Incidence rate, per 1000 PYs | 7.04 (6.38-7.77) | 8.44 (7.58-9.40) | 10.01 (9.06-11.06) | 11.36 (10.78-11.98) | |  |
| Multivariable-adjusted model^※^ | Reference | 1.09 (0.94-1.26) | 0.97 (0.84-1.12) | 0.89 (0.79-1.01) | |  |
| **Diabetes mellitus** |  |  |  |  | | 0.564 |
| No |  |  |  |  | |  |
| N/n | 429/9,962 | 321/6,437 | 376/6,802 | 1,292/22,289 | |  |
| Incidence rate, per 1000 PYs | 4.85 (4.41-5.33) | 5.62 (5.04-6.27) | 6.29 (5.69-6.96) | 6.64 (6.29-7.01) | |  |
| Multivariable-adjusted model | Reference | 0.91 (0.70-1.19) | 0.87 (0.67-1.12) | 0.89 (0.72-1.11) | |  |
| Yes |  |  |  |  | |  |
| N/n | 109/1,286 | 102/937 | 113/1,026 | 494/3,509 | |  |
| Incidence rate, per 1000 PYs | 9.75 (8.08-11.76) | 12.62 (10.39-15.32) | 12.88 (10.71-15.49) | 16.84 (15.42-18.39) | |  |
| Multivariable-adjusted model | Reference | 1.09 (0.94-1.26) | 0.97 (0.84-1.12) | 0.89 (0.79-1.01) | |  |
| **Dyslipidemia** |  |  |  |  | | 0.682 |
| No |  |  |  |  | |  |
| N/n | 291/6,603 | 238/4,707 | 288/4,768 | 1,097/17,306 | |  |
| Incidence rate, per 1000 PYs | 4.96 (4.42-5.57) | 5.70 (5.02-6.48) | 6.88 (6.13-7.72) | 7.27 (6.85-7.72) | |  |
| Multivariable-adjusted model | Reference | 1.01 (0.85-1.20) | 0.96 (8.82-1.14) | 0.87 (0.76-1.00) | |  |
| Yes |  |  |  |  | |  |
| N/n | 247/4,645 | 185/2,667 | 201/3,060 | 689/8,492 | |  |
| Incidence rate, per 1000 PYs | 6.03 (5.32-6.83) | 7.88 (6.82-9.10) | 7.53 (6.56-8.65) | 9.43 (8.75-10.16) | |  |
| Multivariable-adjusted model | Reference | 1.11 (0.92-1.35) | 0.93 (0.77-1.12) | 0.94 (0.80-1.11) | |  |

Abbreviations: HR, hazard ratio; CI, confidence interval; BMI, body mass index; PYs, person-years.

Adjusted for sex, educational level (less than high school, high school/above), body mass index, estimated glomerular filtration rate (<30, 30 ≤eGFR<60, or ≥ 60 ml/min/1.73 m^2^), high-sensitivity C-reactive protein (< 1.0, 1.0 ≤ Hs-CRP ≤ 3.0, or > 3.0 mg/L), low density lipoprotein cholesterol, high density lipoprotein cholesterol, hypertension, diabetes mellitus, and use of antihypertensive, hypoglycemic, and lipid-lowering medications (yes/no for each).
